# Factors Predicting Influenza Vaccination Adherence Among Patients Undergoing Dialysis in Taif City, Saudi Arabia: A Cross-Sectional Study

**DOI:** 10.7759/cureus.40209

**Published:** 2023-06-10

**Authors:** Maha Z Almutairi, Ayman A Atalla

**Affiliations:** 1 Preventive Medicine, Ministry of Health, Taif, SAU; 2 Family Medicine, Taif University, Taif, SAU

**Keywords:** influenza coverage, knowledge, dialysis, vaccination, saudi arabia

## Abstract

Background: Influenza infection can cause severe complications and hospitalization in patients with end-stage renal disease. Despite the importance of influenza vaccination in preventing such complications, adherence to vaccination among these patients is often inadequate.

Objective: To investigate the factors that predict influenza vaccination adherence among patients undergoing in-center dialysis in Taif City, Saudi Arabia.

Methods: Analytical cross-sectional study was conducted in dialysis units of different hospitals in Taif City, Saudi Arabia. A predesigned questionnaire was used for data collection which included questions related to sociodemographic characteristics, knowledge about influenza vaccination, perceived risks of influenza infection, and vaccine-related questions.

Results: A total of 463 individuals were included in the analysis. The median score for knowledge was 6/10, with 60.9% of patients demonstrating good knowledge. In terms of vaccination status, 64.1% had received the influenza vaccine for the current year, with 47.3% adhering to yearly vaccination, 23.1% receiving vaccines irregularly, and 29.6% never receiving the vaccine. Among those who did not receive the vaccine, 21.8% were concerned about the side effects, 15.1% did not believe in the vaccine's effectiveness, and 14.5% were influenced by the media. Adherence to vaccination was significantly associated with good knowledge (OR=2.4), a higher perceived risk of hospitalization (OR=2), and a higher perceived risk of death (OR=2.2).

Conclusion: In conclusion, the study reports predictors that influence influenza vaccine adherence among patients receiving dialysis in Saudi Arabia. Furthermore, the study highlights the importance of knowledge, perceived risk, and healthcare workers' advice in influenza vaccine adherence among patients receiving dialysis.

## Introduction

Chronic kidney disease (CKD) is a complex medical condition characterized by impaired kidney function and the buildup of waste and fluids in the bloodstream [1]. The escalating prevalence of CKD poses a considerable financial burden on healthcare systems worldwide, affecting 11% to 13% of the global population [1]. Among the Saudi population, the incidence and prevalence of CKD have risen significantly in recent decades [2]. The exact number of individuals in each CKD stage in Saudi Arabia remains uncertain despite ongoing research. However, there is a noticeable increase in the occurrence and prevalence of CKD throughout the country, particularly in the Western region due to its diverse population. The primary causative factor of CKD in Saudi Arabia is diabetes mellitus which affects between 12% and 26% of the population, followed by hypertension, which affects around 25% of adults [3].

End-stage renal disease (ESRD) or CKD can lead to immune dysfunction, which can increase susceptibility to infectious diseases. Among individuals with ESRD, infections are a leading cause of mortality and influenza poses a significant risk for severe complications [4]. Dialysis patients have a significantly higher mortality rate for pneumonia and sepsis compared to the general population, with up to a 100-fold increase for the latter [5]. Influenza is a highly contagious respiratory tract infection that can result in significant morbidity and mortality, particularly among vulnerable populations such as the elderly, pregnant women, and those with chronic conditions. Influenza infection can lead to viral pneumonia and subsequent bacterial infection. In the United States, influenza imposes a substantial burden on public health and the economy, with an estimated 225,000 hospitalizations and 36,000 deaths annually, costing approximately $87 billion [6,7].

The Centers for Disease Control and Prevention (CDC) recommends that individuals diagnosed with CKD and ESRD should undergo routine influenza vaccinations to minimize the risks of disease-related complications [8]. Prior research has observed that the administration of influenza vaccines is associated with reduced hospitalization and mortality rates among elderly and high-risk populations. For patients diagnosed with ESRD, the current standard of care is to receive annual influenza vaccinations [7]. Annual vaccination is considered to be the most effective strategy in preventing a significant number of influenza infections, thereby reducing associated morbidity and mortality. Historically, the primary vaccination strategy has focused on immunizing high-risk groups rather than the entire population. The World Health Organization (WHO) advocates the administration of influenza vaccination to individuals aged 65 years or older and those with chronic medical conditions, regardless of age. Such conditions may include chronic pulmonary, cardiovascular, and neurological diseases, hematological disorders, immunosuppression caused by disease or treatment, HIV/AIDS, and neuromuscular conditions, among others [9].

The inadequate vaccination coverage observed among high-risk groups such as health workers, people with a compromised immune system, and people undergoing dialysis is concerning [10]. Therefore, this study aimed to investigate the level of knowledge and behavior related to influenza among patients receiving dialysis services in hospitals located in Taif who are particularly vulnerable to severe complications from influenza infection. Furthermore, the study identified the factors that affect adherence to the seasonal influenza vaccine in this patient group. The goal is to generate new insights that can inform strategies to improve vaccination uptake in this vulnerable population.

## Materials and methods

Study setting

This is an analytical cross-sectional study. The study was conducted in dialysis units of different hospitals in Taif City, Saudi Arabia. All beneficiaries of the dialysis services of Taif were included in the study. All Saudi patients of governmental hospitals of both genders aged ≥18 years were eligible to participate in the study with no specific exclusion criteria applied. No minimum duration of undergoing dialysis was applied to the eligibility criteria.

The sample size was determined using EPI-INFO-7 software. The calculation revealed a minimum required sample of 384 dialysis patients. The equation was built assuming that the percentage of adherence to influenza vaccination is 50%, with a precision of 5%, and a confidence level of 95%. After allowing an additional 20% to account for non-respondents, the final sample size was set at ~460 patients. A stratified sampling technique was used to ensure that the sample would be representative of the target population. Out of four dialysis centers, two were selected randomly. All dialysis patients who had been registered in the registries were divided into two male and female strata. A proportionate random sample was selected from the included centers by gender strata.

Data collection

A predesigned questionnaire was used for the data collection. The validity of the questionnaire was obtained through forward-backward translation. Two bilingual translators, who were native Arabic speakers and proficient in English, conducted forward translation. Afterward, two native English-speaking translators, who were fluent in Arabic but unfamiliar with the scales' concepts, performed backward translation. To ensure that the translation did not compromise the content validity of the questionnaire, the back-translated English version was compared to the original English version, and any inconsistencies were resolved. The data collection was done using face-to-face interviews guided by the predesigned translated questionnaire. 

The questionnaire consisted of three sections. The first section asked about the patients' sociodemographic characteristics. The second section consisted of 10 knowledge questions about the mode of transmission, influenza, vaccine complications, and the use of antibiotics to treat influenza. The third section included questions related to vaccines, such as whether the patient received the current year's influenza vaccine, whether they adhere to getting vaccinated every year, reasons for not getting vaccinated, sources of information, and physician recommendations for vaccination. After calculating the item scores, the median was used as a cut-off point to further classify knowledge into good and poor levels. 

Statistical analysis 

All the statistical analyses were carried out using the Statistical Package for the Social Sciences (IBM Corp. Released 2021. IBM SPSS Statistics for Macintosh, Version 29.0. Armonk, NY: IBM Corp). Tests of normality were applied to continuous variables and were summarized using mean and standard deviation (SD) or median and interquartile range (IQR) as appropriate. Frequency tables and proportions were used to summarize categorical variables. The outcome variable was recoded to be binary as adhered to vaccination every year vs. not adhered. The answers to 10 items measuring knowledge were recoded into correct vs. wrong and summed for a total knowledge score after testing the reliability using Kuder-Richardson (KR-20). The test of the reliability of KR-20 yielded a coefficient of 0.73.

The chi-square test was used to assess the association between categorical variables, while the Fisher-Freeman-Halton exact test was used when more than 20% of cells had an expected count of less than five. Chi-square for trend was used for ordinal independent variables. Variables that were significantly associated with influenza vaccine adherence were further analyzed using multivariate binary logistic regression. P-values <0.05 were considered significant.

Ethical considerations

This study was approved by the research and ethics committee of the King Abdulaziz City for Science and Technology Institutional Review Board (IRB Registration Number: HAP-02-T-067; approval number: 552). All patients were informed about the purpose of the study, and written consent to participate in the study was obtained prior to data collection. No personal identifiers were collected from the data, and the data were used for research purposes only.

## Results

A total of 463 cases were analyzed. Older age groups represented the majority of patients with about one-third (31.5%) in the age group of 55-64 years old and 27.4% in the age group of 65 years and older. Males represented 52.3%, and the majority (62.2%) were married. About one-third (30.5%) were illiterate, and 39.5% were housewives. The patients were asked to give their perspectives on their own risk of hospital admission and death when they contract influenza infection. Out of five, the median and IQR for the perceived risk of hospital admission were (2, 1-3), while the perceived risk of death was lower with a median and IQR of (1, 0-2). The items of knowledge questions were computed to a total score out of 10. The median and IQR for knowledge scores were (6, 4-8). Of the total, 60.9% of the total had a good level of knowledge. The descriptive analysis of demographic characteristics, knowledge, and perceived risks is given in Table 1.

**Table 1:** Respondents’ characteristics, knowledge, and perceived risks regards to influenza in all samples (n=463) and vaccinated patients (n=219)

		All patients	Adherent			
		Frequency (%)	%	OR	95% CI	P-value
Demographic characteristics						
Age	<65 Years	336 (72.6)	46.1			0.412
	≥65 Years	127 (27.4)	50.4	1.2	0.8, 1.8	
Gender	Male	242 (52.3)	47.5	1	0.7, 1.5	0.921
	Female	221 (47.7)	47.1			
Education	Less than high school	278 (60)	48.9	1.2	0.8, 1.7	0.392
	High school and above	185 (40)	44.9			
Knowledge and perceived risk						
Knowledge	Poor	181 (39.1)	34.3			<0.001
	Good	282 (60.9)	55.7	2.4	1.6, 3.6	
Hospitalization	<3 (0-5 scale)	287 (62)	40.8			<0.001
	≥3 (0-5 scale)	176 (38)	58	2	1.4, 2.9	
Death	<3 (0-5 scale)	357 (77.1)	42.9			<0.001
	≥3 (0-5 scale)	106 (22.9)	62.3	2.2	1.4, 3.4	

About two-thirds (64.1%) took the influenza vaccine for the current year. About half (47.3%) have adhered to the vaccine yearly, 23.1% indicated irregular vaccination, and 29.6% indicated never receiving the influenza vaccine. Those who did not receive the vaccine in the year 2022 were asked about the reason; 21.8% indicated fear of the side effects, 15.1% did not believe the vaccine is effective, and 14.5% were afraid that the vaccine would complicate their condition. Media such as TV and radio were the most common source of information among 75.4%, followed by social media by 54%. Adherence to vaccination had significantly higher odds among those with good knowledge (OR=2.4), higher perceived risk of hospitalization (OR=2), and among those with higher perceived risk of death (OR=2.2). The results are presented in Table 1.

While 82.3% indicated that their nephrologists has recommended taking the influenza vaccine, only 38.2% indicated that their general doctor has recommended the vaccination against influenza. Few patients have asked their general doctor, nephrologist, and other healthcare workers (HCWs) regarding the influenza vaccine (21.8%, 24.6%, and 10.6%), respectively. Vaccination adherence had significantly higher odds among the groups who asked their doctor, nephrologist, or HCW. Furthermore, vaccine-adherent patients had higher odds of consulting TV and radio, brochures/posters, and receiving a recommendation from their general doctor (OR= 2, 1.8, and 2.7), respectively. Those findings were statistically significant (Table 2).

**Table 2:** Persons and media consulted by patients on influenza vaccination in all samples (n=463) and vaccinated patients (n=219)

		All patients	Adherent			
		Frequency (%)	%	OR	95% CI	P-value
Person asked by patients for information on vaccine						
General doctor	Asked	101 (21.8)	70.3	3.4	2.1, 5.5	<0.001
	Not asked	362 (78.2)	40.9			
Nephrologist	Asked	114 (24.6)	71.1	3.8	2.4, 5.9	<0.001
	Not asked	349 (75.4)	39.5			
Other healthcare workers	Asked	49 (10.6)	75.5	3.9	1.9, 7.8	<0.001
	Not asked	414 (89.4)	44			
Family, friends, co-workers	Asked	39 (8.4)	48.7	1.1	0.6, 2.1	0.853
	Not asked	424 (91.6)	47.2			
Patients with similar condition	Asked	28 (6)	53.6	1.3	0.6, 2.8	0.493
	Not asked	435 (94)	46.9			
At least one	Asked	175 (37.8)	63.4	0.3	0.2, 0.5	<0.001
	Not asked	288 (62.2)	37.5			
Media consulted by patient about vaccine						
Media such as TV and radio	Consulted	349 (75.4)	43			0.001
	No	114 (24.6)	60.5	2.0	1.3, 3.1	
News	Consulted	87 (18.8)	56.3	1.6	0.9, 2.5	0.061
	No	376 (81.2)	45.2			
Brochures/posters	Consulted	142 (30.7)	57.7	1.8	1.2, 2.7	0.003
	No	321 (69.3)	42.7			
Internet	Consulted	236 (51)	43.2			0.073
	No	227 (49)	51.5	1.4	0.9, 2	
Social media	Consulted	250 (54)	44.4			0.176
	No	213 (46)	50.7	1.3	0.9, 1.9	
Recommendation of vaccine by						
General doctor	Yes	177 (38.2)	62.1			<0.001
	No	286 (61.8)	38.1	2.7	1.8, 3.9	
Nephrologist	Yes	381 (82.3)	46.7	1.1	0.7, 1.8	0.589
	No	82 (17.7)	50			

Variables that were significantly associated with influenza vaccine adherence were further analyzed using multivariate binary logistic regression to identify the significant predictors of vaccine adherence collectively. Good knowledge, higher perceived risk of death, asking healthcare workers (including general physicians and nephrologists), consulting TV and radio, and vaccine recommendations by general doctors were all significant predictors of backward stepwise regression analysis. The results are shown in Table 3.

**Table 3:** Multivariate predictors of influenza vaccination adherence among patients receiving dialysis

	Logistic regression		Stepwise regression		
	OR (95% CI)	P-value	Beta	OR (95% CI)	P-value
Good knowledge	2.2 (1.4, 3.4)	0.001	0.73	2.1 (1.4, 3.2)	0.001
Perceived risk of hospitalization	1.1 (0.6, 1.9)	0.935			
Perceived risk of death	2.6 (1.4, 5.2)	0.006	0.95	2.6 (1.6, 4.3)	<0.001
Asked general doctor	2 (1.1, 4)	0.044	0.79	2.3 (1.2, 4.3)	0.019
Asked nephrologist	1.6 (0.9, 3)	0.132	0.67	2 (1.2, 3.4)	0.018
Asked other healthcare worker	1.9 (0.9, 4.3)	0.136			
Consulted TV and radio	2.5 (1.4, 3.3)	<0.001	-1.08	2.5 (1.7, 3.3)	<0.001
Consulted brochures	1.2 (0.8, 1.9)	0.590			
Vaccine recommended by a general doctor	0.6 (0.4, 0.9)	0.010	-0.67	0.6 (0.4, 0.9)	0.011

## Discussion

This study represents the first extensive inquiry into the understanding and behaviors pertaining to the influenza vaccine among individuals receiving dialysis treatment in Saudi Arabia. The objective of the study was to determine the factors that forecast adherence to influenza vaccination among this population. A similar study was carried out in Italy by Battistella et al., who documented deficient compliance with influenza vaccination and below-par levels of awareness concerning influenza infection among individuals at heightened risk of unfavorable outcomes [11]. According to their findings, the adherence rate to influenza vaccination among patients receiving dialysis across multiple centers was 57.5%. Furthermore, a considerable proportion of participants (47.5%) believed that the influenza vaccine could result in influenza infection, while 45.7% considered antibiotics to be a viable treatment option for influenza [11]. Our study's results were consistent with these findings, revealing that 64.1% of dialysis patients received the influenza vaccine, with 47.3% maintaining annual adherence and 29.6% never having received the vaccine. However, our vaccination rates were marginally higher than those of a previous study conducted in Taif, Saudi Arabia, which reported an adherence rate of 43.5% among diabetic patients [12]. The difference in vaccination adherence between dialysis and diabetic patients can be attributed to the frequency of follow-up and hospital visits. Dialysis patients visit the hospital several times per week and are more likely to be offered the influenza vaccine. In contrast, diabetic patients may have follow-up intervals of up to three months. Our study results align with a study conducted in London, which showed a 67% vaccination rate in dialysis patients [13]. According to the survey by Gawryś et al., 45% of the participants reported receiving annual influenza vaccinations and 87.4% expressed the belief that the vaccinations were effective [14]. Furthermore, they found that positive belief in the efficacy of the influenza vaccine was significantly associated with the frequency of regular vaccinations (P<0.01). Individuals who reported irregular or no vaccinations cited fear of adverse events (29.2%), belief in vaccine inefficacy (26.4%), and lack of vaccine-related information (22.6%) as the reasons for their decision [14]. These findings were in-line with our results which found fear of adverse reactions as a major factor for vaccine hesitancy.

In line with Battistella et al., our findings showed that TV and radio were the most common source of information among 75.4%, followed by social media by 54% [11]. The role of media in increasing adherence to the influenza vaccine has been reported in previous studies as well [15,16]. As seen in the present study, better knowledge about safety, efficacious, and awareness about seasonal vaccination had a higher rate of vaccination adherence [17]. Similar to our findings, a previous study by Alhatim et al. has reported good knowledge regarding vaccination among the Saudi general population [18]. Their results showed that the majority of participants had good knowledge regarding seasonal flu (64.5%) and the flu vaccine (73.3%). However, only 52% of participants had a positive attitude score toward the seasonal influenza vaccination [18].

Our present study has revealed a notable escalation in the likelihood of vaccination adherence within cohorts who sought advice from medical professionals such as doctors, nephrologists, or HCWs. This finding aligns with previous research, which has underscored the pivotal role of HCWs in endorsing influenza vaccination and shaping patients' attitudes toward immunization [12,19]. HCWs are a valuable source of patient information and should be the primary focus of influenza prevention efforts. According to a review, an appeal by the physician to patients to get vaccinated for influenza resulted in an increase of 8.3% compared to the control group [20]. Furthermore, an intervention aimed at encouraging vaccination among patients by a health insurance fund led to a significant increase of 3.2% in vaccination rates (P<0.001) [20]. This highlights the crucial role of healthcare workers in promoting influenza vaccination among dialysis patients. Therefore, healthcare workers should be encouraged to promote influenza vaccination and provide patients with accurate information about the vaccine's benefits.

The present study has a few limitations that need to be considered when interpreting the results. First, the study relied on self-reported data which may be subject to social desirability bias. Secondly, the study did not include patients' cultural beliefs and attitudes toward vaccination which may influence vaccine adherence. Therefore, future research should explore the role of cultural beliefs and attitudes toward influenza vaccination among dialysis patients as they play a significant role in vaccine adherence [21].

## Conclusions

This study provides valuable insights into the factors that influence adherence to influenza vaccination among patients undergoing dialysis in Taif City, Saudi Arabia. The study found that participants had good knowledge about vaccination, which is a positive indicator of their willingness to get vaccinated. Despite having good knowledge, the level of adherence to immunization was not optimal. The study suggests that enhancing communication between healthcare workers including general doctors, nephrologists, and patients could be a potential solution to improve vaccination adherence. This could be achieved by providing patients with accurate information about the benefits of vaccination, addressing their concerns and fears, and creating awareness campaigns to educate patients and healthcare workers about the importance of vaccination. Further research is recommended to validate the findings of this study and identify other factors that may influence vaccination adherence among patients undergoing dialysis. This could include exploring the role of patient education, cultural beliefs, and socioeconomic factors in vaccination adherence. By better understanding the factors that influence vaccination adherence, healthcare providers can develop targeted interventions to improve vaccination rates and ultimately reduce the incidence of influenza-related complications among dialysis patients.
